# Supplementary material for: Comparing serial X-ray crystallography and microcrystal electron diffraction (MicroED) as methods for routine structure determination from small macromolecular crystals
Source: IUCrJ. 2020 Feb 26;7(Pt 2):306–23. doi: 10.1107/S205225252000072X (PMC7055375; doi:10.1107/S205225252000072X)
Supplement: Supplementary file 1 [file m-07-00306-sup1.pdf]

IUCrJ

Volume 7 (2020)

Supporting information for article:

Comparing serial X-ray crystallography and microcrystal electron diffraction (MicroED) as methods for routine structure determination from small macromolecular crystals

Alexander M. Wolff, Iris D. Young, Raymond G. Sierra, Aaron S. Brewster, Michael W. Martynowycz, Eriko Nango, Michihiro Sugahara, Takanori Nakane, Kazutaka Ito, Andrew Aquila, Asmit Bhowmick, Justin T. Biel, Sergio Carbajo, Aina E. Cohen, Saul Cortez, Ana Gonzalez, Tomoya Hino, Dohyun Im, Jake D. Koralek, Minoru Kubo, Tomas S. Lazarou, Takashi Nomura, Shigeki Owada, Avi J. Samelson, Tomoyuki Tanaka, Rie Tanaka, Erin M. Thompson, Henry van den Bedem, Rahel A. Woldeyes, Fumiaki Yumoto, Wei Zhao, Kensuke Tono, Sebastien Boutet, So Iwata, Tamir Gonen, Nicholas K. Sauter, James S. Fraser and Michael C. Thompson

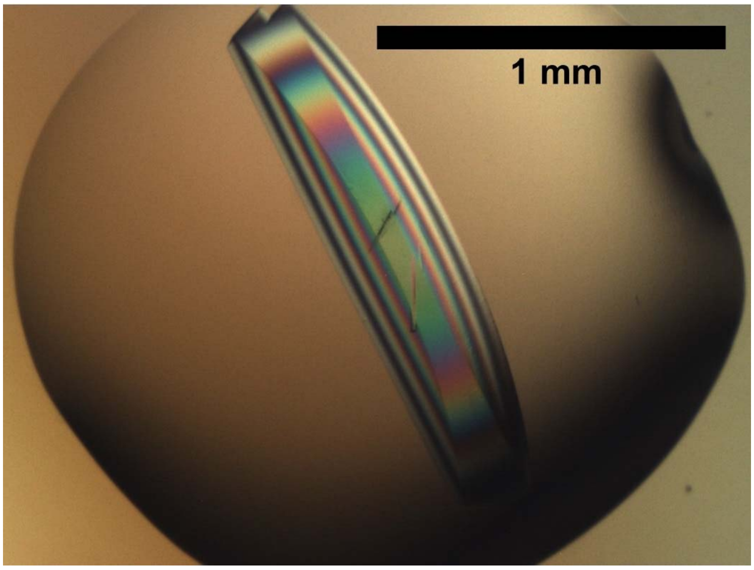

Figure S1 Previously, CypA crystallization conditions were optimized to produce very large single crystals. The pictured crystal (viewed using a cross-polarizer) was over 1 mm long and was visible to the naked eye.

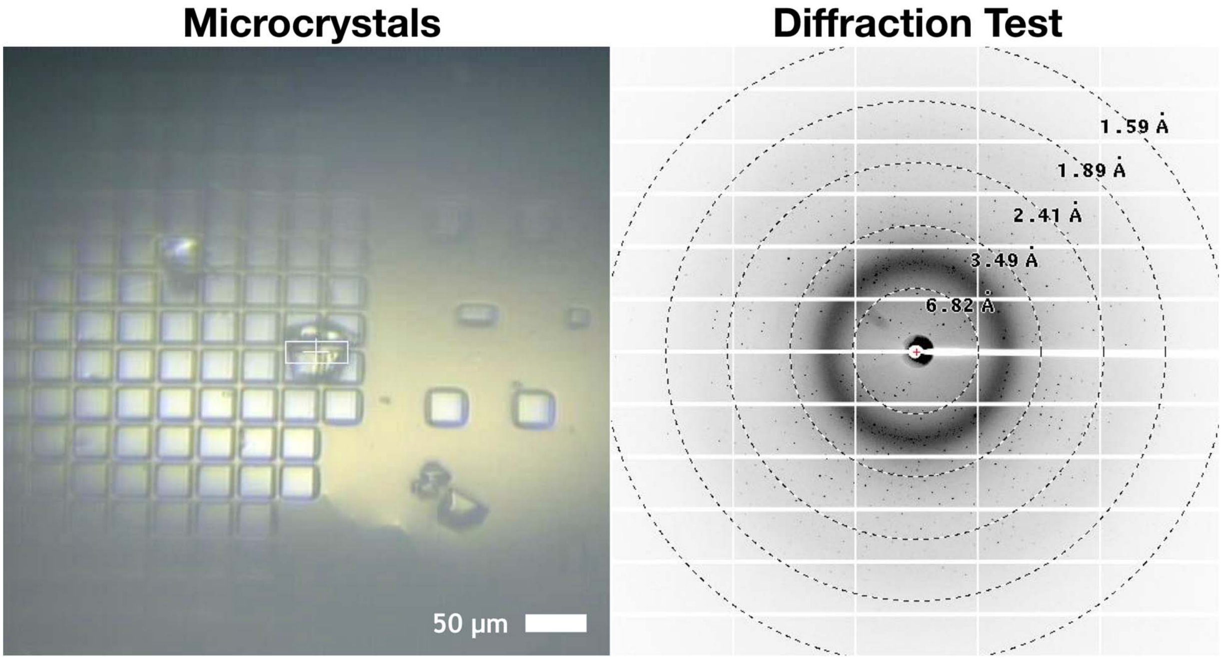

Figure S2 Image of microcrystals on a Mitegen micromesh grid (left) and the subsequent diffraction from one of these crystals (right). Crystals were 20-50 µm in all dimensions, and diffracted to the edge of the detector at 1.59 Å.

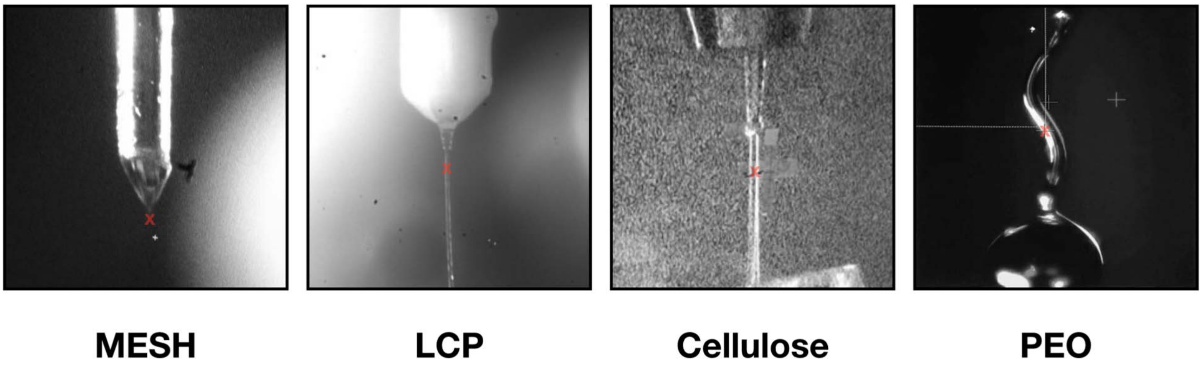

Figure S3 Images of jets delivering microcrystal slurries to the XFEL interaction point (red “x” in each image). Minimal viscogens were added to the crystalline slurry for the MESH injector system. When using a viscous extrusion type of injector, a variety of carrier media were tested, including LCP, Cellulose, and PEO.

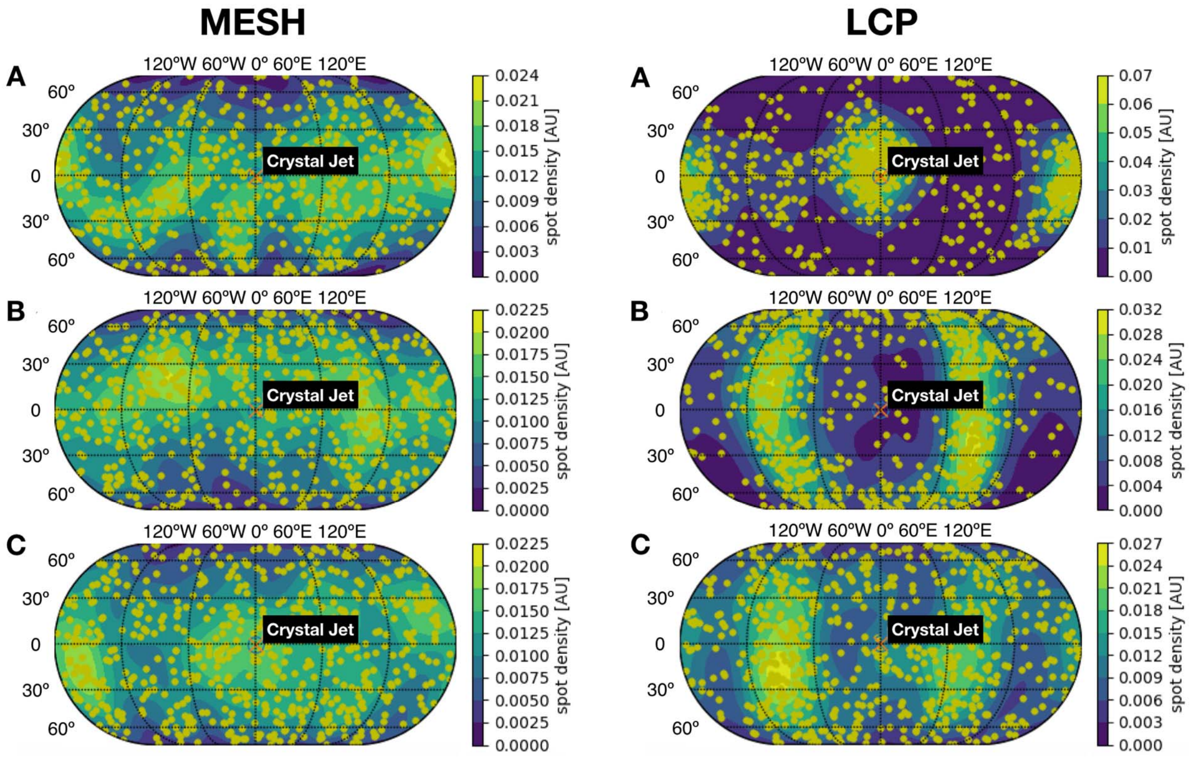

Figure S4 Maps of crystal axis (A, B, C) orientations from the MESH data collection (left panel) and from the LCP data collection (right panel). Axis orientations (in polar coordinates) for individual crystals are depicted as yellow spots, and the background color reflects the frequency with which a particular orientation was observed. A subset of each dataset is shown for visual clarity. The orientations appear evenly distributed for the MESH data, with no major bias introduced by the electric field created by the injection system. The viscosity of the LCP carrier media appears to have induced an orientation bias, but did not prohibit collection of a complete dataset with high redundancy.

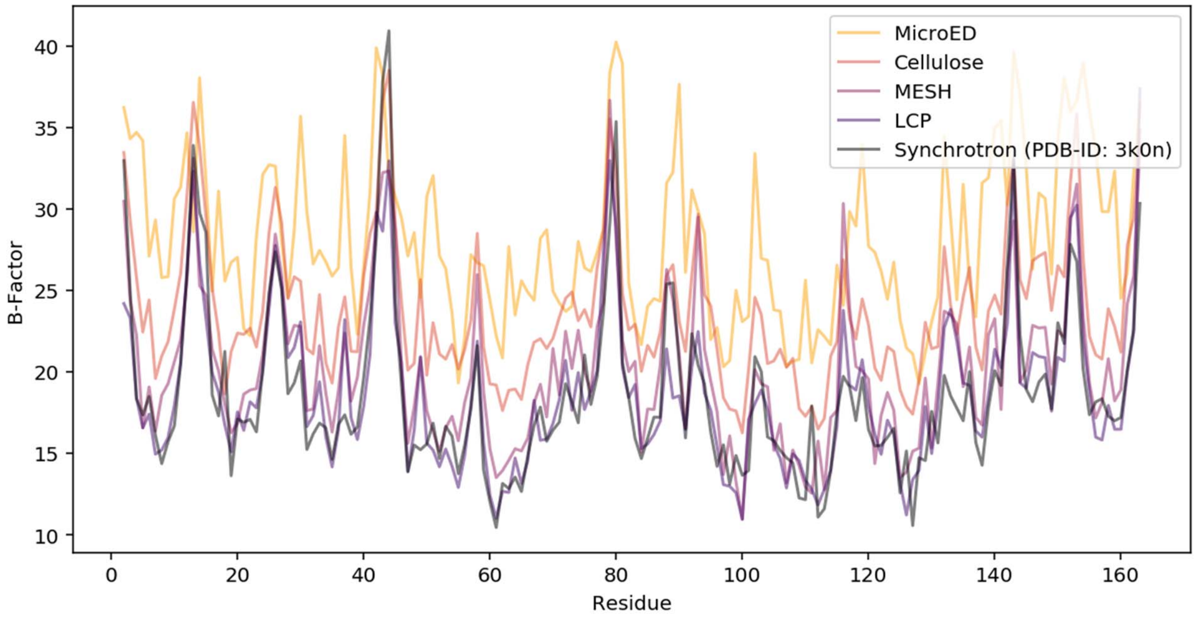

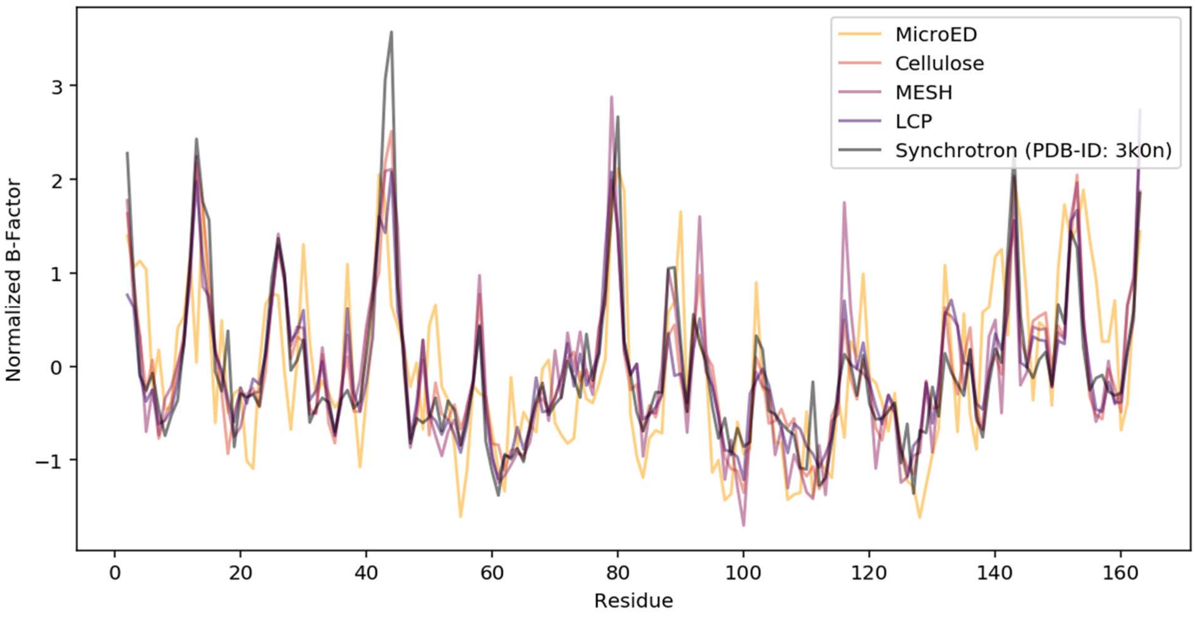

Figure S5 Raw (top) and normalized (bottom) B-factor per residue across data sets. A previously published structure, solved at room temperature using rotational collection from a single crystal (PDB ID *3K0N*) is provided for reference. Most variation is systematic, and thus is removed by normalization.

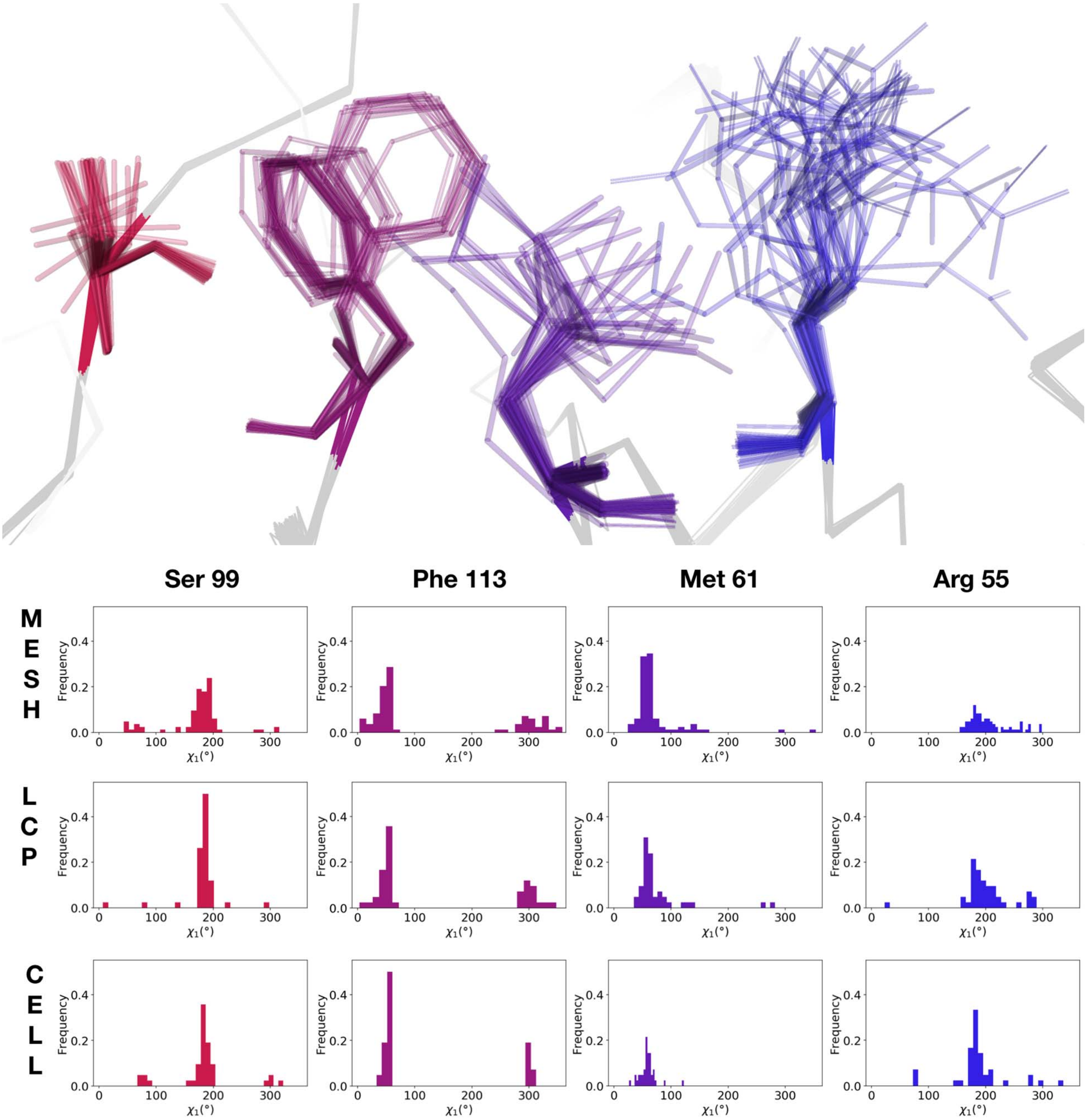

Figure S6 Visualization of the ensemble of conformers generated via phenix.ensemble_refine for the three serial XFEL datasets. The analysis focuses on a network of amino acid side chains that are known to be dynamic and important for function. In the top panel, the ensemble model for the cellulose dataset is displayed. Sticks are shown for the residues of interest (R55, M61, S99, F113), while the backbone is displayed as a ribbon for the rest of the structure. In the bottom panel, histograms of the distribution of chi1 angles are plotted for each of the four residues from the respective ensemble. Pairwise chi-square analysis revealed no significant differences between the rotamer distributions.

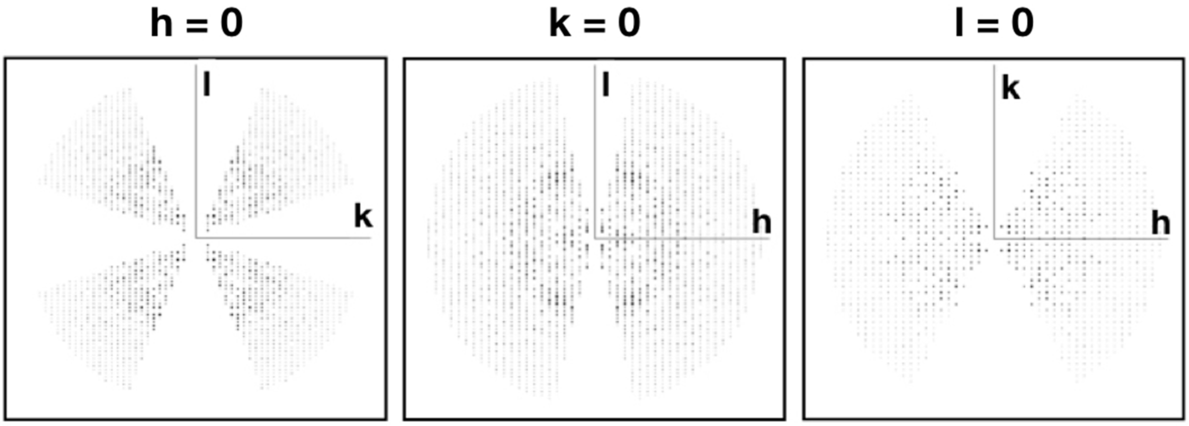

Figure S7 MicroED data visualized on two-dimensional slices of the reciprocal lattice. Measured reflections are visualized in black. Missing measurements along the kl plane may contribute to challenges in assignment of unit cell dimensions.

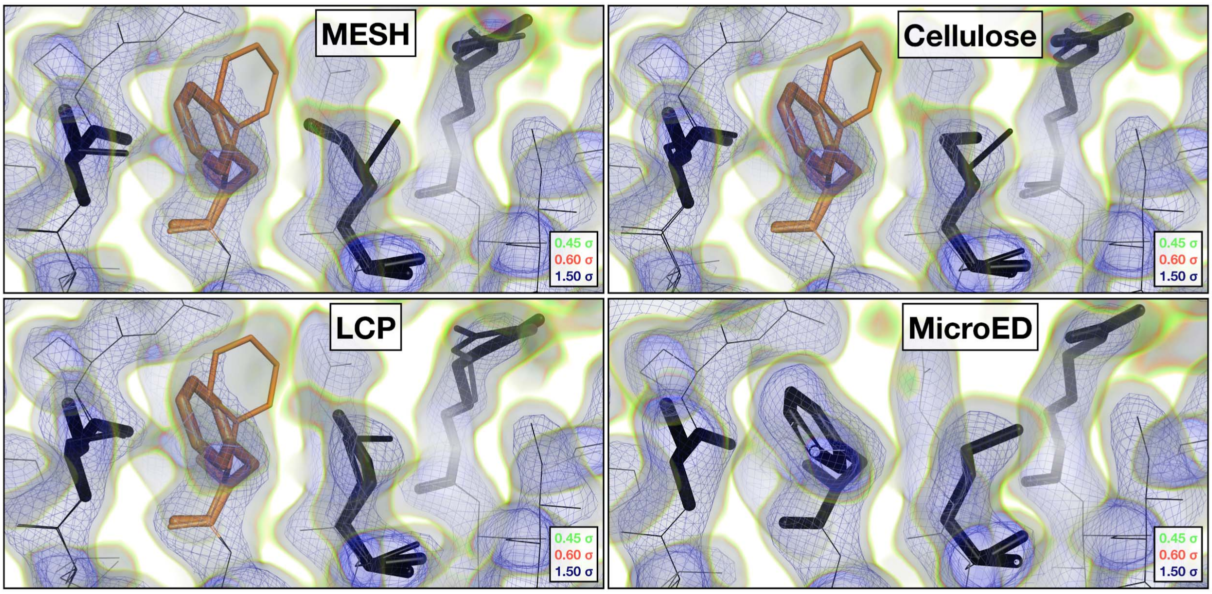

Figure S8 Visualization of all four datasets truncated at 2.5 Å. Comparison of the 2mFoFc maps and refined multi-conformer models reveals evidence for alternative conformations in the room temperature data (MESH, LCP, Cellulose), while cryogenic data (MicroED) supports a single conformer model.

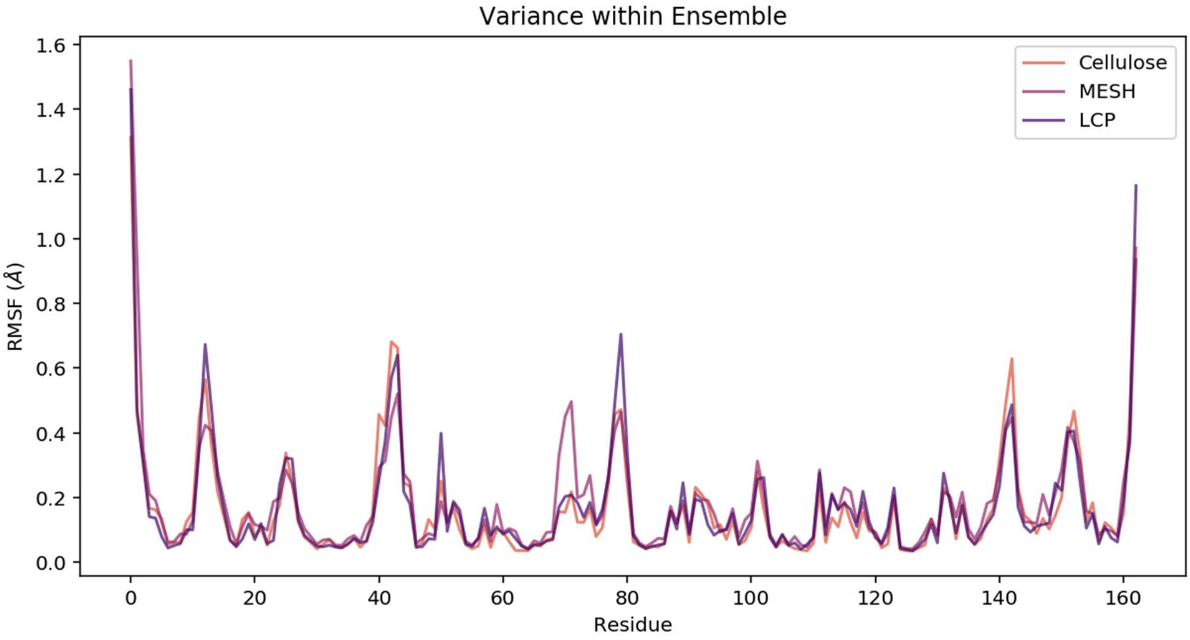

Figure S9 Average RMSF per residue for each ensemble generated for the serial XFEL datasets. A loop containing residues 64-74 samples more conformations in the MESH ensemble than in the other two.

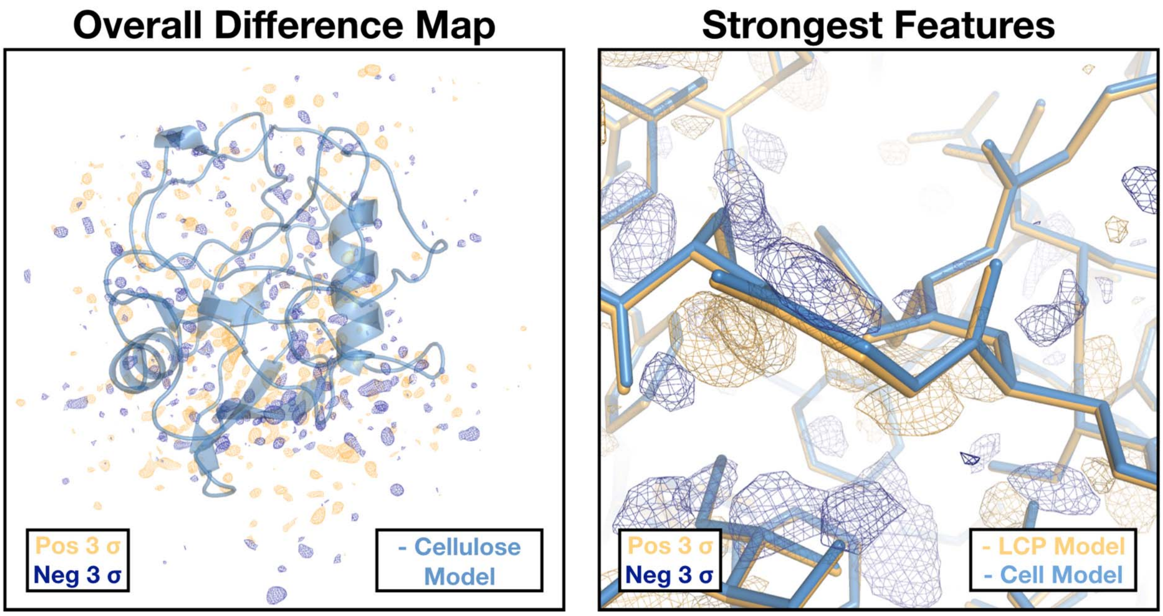

Figure S10 Visualization of an Fo-Fo difference map, displayed at 3 sigma, for the LCP and Cellulose datasets. In the left panel the overall difference map is shown, with the Cellulose model visualized for structural context. In the right panel the strongest features within the map are visualized, with both the LCP model and the Cellulose (Cell) model visualized for comparison.

Table S1 Results of data indexing with XDS and unit cell refinement with REFMAC5.

Unit cell axis	a	b	c
XDS indexed (merged)	42.40	53.40	87.76
XDS indexed (std. dev.)	0.05	0.03	0.06
REFMAC5 refined	42.56	53.56	88.07

Table S2 Quantitative comparison of unit cell distributions across XFEL datasets

One-way ANOVA with post-hoc Tukey HSD tests revealed that all three datasets were significantly different across all three edges of the crystalline unit cell, as denoted by the respective *p*-values for each test

	A axis	B axis	C axis
MESH Unit Cell (N = 18358)	43.32±0.11 Å	52.94±0.09 Å	89.87±0.21 Å
LCP Unit Cell (N = 11821)	43.10±0.18 Å	52.65±0.13 Å	89.29±0.26 Å
Cellulose Unit Cell (N = 23947)	43.00±0.26 Å	52.60±0.23 Å	89.20±0.37 Å
ANOVA	$p < 0.0001$	$p < 0.0001$	$p < 0.0001$
MESH vs. LCP Tukey HSD Post-hoc Test	Difference = -0.22<br>95 % CI = -0.2256 to -0.2144<br>$p < 0.0001$	Difference = -0.29<br>95 % CI = -0.2948 to -0.2852<br>$p < 0.0001$	Difference = -0.58<br>95 % CI = -0.5883 to -0.5717<br>$p < 0.0001$
MESH vs. Cellulose Tukey HSD Post-hoc Test	Difference = -0.32<br>95 % CI = -0.3247 to -0.3153<br>$p < 0.0001$	Difference = -0.34<br>95 % CI = -0.3440 to -0.3360<br>$p < 0.0001$	Difference = -0.67<br>95 % CI = -0.6769 to -0.6631<br>$p < 0.0001$
LCP vs. Cellulose Tukey HSD Post-hoc Test	Difference = -0.10<br>95 % CI = -0.1053 to -0.0947<br>$p < 0.0001$	Difference = -0.05<br>95 % CI = -0.0545 to -0.0455<br>$p < 0.0001$	Difference = -0.09<br>95 % CI = -0.0979 to -0.0821<br>$p < 0.0001$

.
